# Systemic immune-inflammation index predicts the clinical outcome in patients with metastatic renal cell cancer treated with sunitinib

**DOI:** 10.18632/oncotarget.10515

**Published:** 2016-07-09

**Authors:** Cristian Lolli, Umberto Basso, Lisa Derosa, Emanuela Scarpi, Teodoro Sava, Matteo Santoni, Simon J. Crabb, Francesco Massari, Michele Aieta, Vincenza Conteduca, Marco Maruzzo, Francesca La Russa, Matthew Wheater, Rossana Berardi, Luca Galli, Ugo De Giorgi

**Affiliations:** ^1^ Department of Medical Oncology, Istituto Scientifico Romagnolo per lo Studio e la Cura dei Tumori (IRST) IRCCS, Meldola, Italy; ^2^ Medical Oncology Unit 1, Department of Clinical and Experimental Oncology, Istituto Oncologico Veneto IOV IRCCS, Padova, Italy; ^3^ Oncology Unit 2, University Hospital of Pisa, Pisa, Italy; ^4^ Department of Medical Oncology, Azienda Ospedaliera Universitaria Integrata Verona, Verona, Italy; ^5^ Department of Medical Oncology, Polytechnic University of the Marche Region, Azienda Ospedaliero-Universitaria, Ospedali Riuniti Umberto I-GM Lancisi and G Salesi, Ancona, Italy; ^6^ Department of Medical Oncology, University Hospital Southampton NHS Foundation Trust, Southampton General Hospital, Southampton, UK; ^7^ Department of Medical Oncology, IRCCS CROB Centro di Riferimento Oncologico della Basilicata, Rionero in Vulture, Italy; ^8^ Present address: Division of Oncology, S.Orsola-Malpighi Hospital, Bologna, Italy

**Keywords:** systemic immune inflammation index, renal cell carcinoma, RCC, prognostic factor, sunitinib

## Abstract

**Background:**

In this retrospective analysis, we explored the prognostic and predictive value of the systemic immune-inflammation index (SII), based on lymphocyte, neutrophil, and platelet counts, at baseline and changes at week 6 during first-line sunitinib in patients with metastatic renal cell cancer (RCC).

**Results:**

Patients were stratified into high SII (≥ 730) and low SII (< 730) groups. SII was associated with objective response, *p* < 0.0001. The median PFS was 6.3 months (95% CI 5.5–8.9) in patients with SII ≥ 730 and 18.7 months (95% CI 14.7–22.8) in those with SII < 730, *p* < 0.0001. The median OS was 43.6 months (95% CI 35.3–52.1) in patients with SII < 730, and 13.5 months (95% CI 9.8–18.5) in those with SII ≥ 730, *p* < 0.0001. In multivariate analysis, performance status, IMDC score and SII were able to predict OS (HR = 3.29, HR = 1.71 and HR = 1.79, respectively).

**Materials and Methods:**

We included 335 consecutive RCC patients treated with first-line sunitinib. The X-tile 3.6.1 software (Yale University, New Haven, CT) was used for bioinformatic analysis of the data to determine the cutoff value of SII. Progression-free survival (PFS), overall survival (OS) and their 95% confidence interval (95% CI) were estimated by Kaplan-Meier method and compared with logrank test. The impact of SII conversion at week 6 of treatment on PFS and OS was evaluated by Cox regression analyses.

**Conclusions:**

The SII and its changes during treatment represent a powerful prognostic indicator of clinical outcome in patients with metastatic RCC.

## INTRODUCTION

Renal cell carcinoma (RCC) is the most common type of kidney cancer in adults and about 30% of patient with diagnosis of kidney cancer develop metastatic disease [1]. A predominant role in kidney cancer is played by inactivation of Von Hippen Lindau (VHL) tumor suppressor gene with consequent increased cellular amount of Hypoxia-Inducible Factor-1 alpha (HIF-1a) that cause abnormal cellular growth and angiogenesis [2–4]. Therefore inhibition of angiogenesis represents the mainstay of treatment of metastatic RCC [5, 6]. Many evidences about the role of host inflammatory response in carcinogenesis and disease progression of many cancers have recently emerged. Proinflammatory cytokines but also immune-inflammatory circulating cells (neutrophils, lymphocytes and platelets) seem to play a role in promoting cancer cell proliferation and invasion [7]. In this scenario inflammatory circulating cells has been recently evaluated and associated with prognosis in several cancers including RCC [8–13].

A new inflammatory index, the systemic immune inflammation index (SII), based on neutrophil, lymphocyte and platelet counts has been recently found to be associated with poor outcome in patients with hepatocellular carcinoma [14]. The rationale of this new index is based on the combination of three factors independently related to prognosis in some cancers. This combination was thought to have a stronger prognostic power.

In this retrospective analysis, we aimed to evaluate prognostic implications of SII at baseline and changes at week 6 during first-line sunitinib in patients with mRCC.

## RESULTS

### Patients

A total of 335 patients with a median age of 63 years (range, 27 to 88 years) who were diagnosed with unresectable or metastatic RCC and underwent first-line treatment with sunitinib were included in the study. Histotype clear cell carcinoma was reported in 94% of cases; among all patients 35%, 52.5% and 12.5% were classified in the favorable, intermediate and poor prognostic groups according to the “International Metastatic Renal Cell Carcinoma Database Consortium (IMDC) model”, respectively. Baseline characteristics of patients are shown in Table 1. An optimal cutoff point for the SII of 730 × 10^9^ stratified these patients into high (≥ 730) and low SII (< 730) groups. Among the study population, 209 and 126 had low and high SII values, respectively.

**Table 1:** Patients' characteristics (*n* = 335)

	No (%)
**Median age, years**(range)	63 (27–88)
**Gender**	
Males	238 (71.0)
Females	97 (29.0)
**ECOG Performance status**	
0	197 (59.2)
1	111 (33.3)
≥ 2	25 (7.5)
missing	2
**Histotype**	
Clear cell carcinoma	315 (94.0)
Papillary	14 (4.2)
Others	6 (1.8)
**MSKCC score**	
Good	98 (29.2)
Intermediate	199 (59.4)
Poor	38 (11.3)
**IMDC score**	
Good	117 (35.0)
Intermediate	176 (52.5)
Poor	42 (12.5)

Abbreviations: ECOG, Eastern Cooperative Oncology Group; MSKCC, Memorial Sloan Kettering Cancer Center; IMDC, International Metastatic Renal Cell Carcinoma Database Consortium.

### SII and objective response

An objective tumor response was reported in 115 of 321 evaluable patients (35.8%), including complete response (CR, *n* = 13, 4.0%) and partial response (PR, *n* = 102, 31.8%), respectively; stable disease (SD) was reported in 141 cases (43.9%) and progressive disease (PD) in 65 (20.1%), whereas in the remaining 14 cases (4.2%) the objective response was not evaluated, mainly due to early clinical deterioration. An association was observed between baseline SII < 730 or ≥ 730 and either objective response (CR+PR vs SD+PD), *p* < 0.0001, or clinical benefit (CR*PR*SD vs PD), *p* < 0.0001, and between 6-week SII < 730 or ≥ 730 and either objective response (CR+PR vs SD+PD), *p* < 0.0001, or clinical benefit (CR*PR*SD vs PD), *p* < 0.0001.

Grade 3–4 toxicities occurred in 162 of 335 (48.4%) patients. Grade 3–4 neutropenia was reported in 24 (7.5%) patients, grade 3–4 thrombocytopenia in 26 (7.8%) and grade 3–4 anaemia in 17 (5%). No correlation between baseline and week-6 SII and grade 3–4 toxicities was found.

### SII and survival

**Figure 1:**
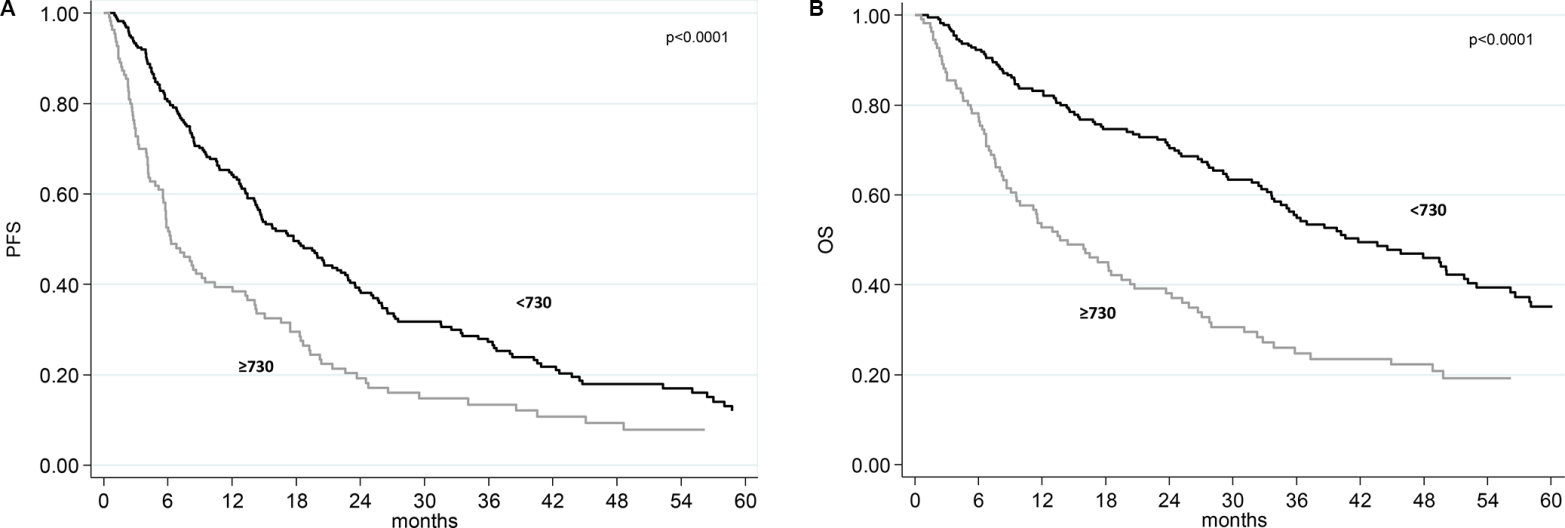
Progression-free survival (PFS) and overall survival (OS) according baseline SII (**A**) Kaplan-Meier plots illustrating PFS according to baseline SII. (**B**) Kaplan-Meier plots illustrating OS according to baseline SII.

The median follow-up was 49 months (range 1 to 102). At the time of analysis, 260 of the 335 (77.6%) patients had progressed and 193 (57.6%) died. The median progression-free survival (PFS) was 14.2 months (95% confidence interval (CI) 12.1–17.2) and the median overall survival (OS) was 32.7 months (95% CI 27.1–36.4). The median PFS was 6.3 months (95% CI 5.5–8.9) in patients with baseline SII ≥ 730 and 18.7 months (95% CI 14.7–22.8) in those with SII < 730, *p* < 0.0001 (Figure 1A). The median OS was 43.6 months (95% CI 35.3-52.1) in patients with baseline SII < 730, and 13.5 months (95% CI 9.8–18.5) in those with baseline SII ≥ 730, *p* < 0.0001 (Figure 1B).

A univariate analysis revealed that ECOG performance status, IMDC score and baseline SII were significant predictors of PFS and OS (Table 2). In multivariate analysis, ECOG performance status and SII at baseline remained significant predictors of PFS (HR = 3.29, 95% CI 2.13–5.07, *p* < 0.0001; HR = 1.71, 95% CI 1.33–2.21, *p* < 0.0001) and of OS (HR = 3.34, 95% CI 2.10–5.23, *p* < 0.0001; HR = 1.84, 95% CI 1.35–2.50, *p* < 0.0003); whereas IMDC score (poor and intermediate vs good risk)s howed a borderline ability to predict PFS (HR = 1.32, 95% CI 0.99–1.76, *p* = 0.058), and remained as predictor of OS only (HR = 1.79, 95% CI 1.25–2.55, *p* = 0.001)

**Table 2:** Univariate analysis for progression-free survival and overall survival

			Progression-free survival				Overall survival		
	n. pts	n. events	Median PFS (months) (95% CI)	HR (95% CI)	*p*	n. events	Median OS (months) (95% CI)	HR (95% CI)	*p*
**Overall**	335	260	14.2 (12.1–17.2)	-		193	32.7 (27.1–36.4)	-	
**Age**									
< 63 years	157	123	14.4 (10.8–18.7)	1.00		83	34.5 (29.1–50.1)	1.00	
≥ 63 years	178	137	14.2 (10.4–17.2)	1.12 (0.88–1.43)	0.353	110	27.1 (20.3–35.7)	1.31 (0.99–1.75)	0.061
**ECOG PS**									
0–1	309	236	14.9 (13.3–18.4)	1.00		171	33.9 (29.1–40.6)	1.00	
2	25	24	3.0 (2.4–5.6)	3.63 (2.37–5.57)	< 0.0001	22	6.5 (2.7–9.6)	4.50 (2.86–7.09)	< 0.0001
**Histotype**									
Clear cell	315	243	14.3 (12.4–17.4)	1.00		179	33.6 (27.8–37.3)	1.00	
Papillary	14	11	7.1 (3.9–31.5)	1.36 (0.74–2.49)	0.321	9	13.3 (4.5-nr)	1.58 (0.81–3.10)	0.180
Other	6	6	10.4 (1.1–22.6)	2.00 (0.89–4.51)	0.094	5	12.4 (1.7-nr)	1.90 (0.78–4.63)	0.159
**MSKCC score**									
Good	98	70	21.4 (17.7–27.5)	1.00		35	63.9 (44.5–75.2)	1.00	
Intermediate	199	161	12.1 (9.2–15.8)	1.48 (1.12–1.97)	0.006	134	27.1 (19.5–32.8)	2.44 (1.68–3.55)	< 0.0001
Poor	38	29	5.7 (3.9–8.3)	2.00 (1.29–3.09)	0.002	24	8.7 (5.4–25.1)	4.04 (2.39–6.82)	< 0.0001
Good	98	70	21.4 (17.7–27.5)	1.00		35	63.9 (44.5–75.2)	1.00	
Intermediate+Poor	237	190	10.6 (8.2–13.4)	1.55 (1.17–2.03)	0.002	158	23.8 (16.8–28.1)	2.59 (1.79–3.74)	< 0.0001
**IMDC score**									
Good	117	84	21.4 (17.2–26.5)	1.00		48	56.6 (38.6–75.2)	1.00	
Intermediate	176	139	13.3 (9.2–17.4)	1.43 (1.09–1.87)	0.010	109	29.4 (24.0–36.0)	1.99 (1.41–2.80)	< 0.0001
Poor	42	37	4.0 (2.7–5.2)	5.09 (3.39–7.66)	< 0.0001	36	5.3 (3.7–8.0)	11.12 (6.95–17.80)	< 0.0001
Good	117	84	21.4 (17.2–26.5)	1.00		48	56.6 (38.6–75.2)	1.00	
Intermediate+Poor	218	176	9.4 (7.5–12.4)	1.65 (1.27–2.15)	0.0002	145	23.7 (14.6–28.1)	2.42 (1.74–3.36)	< 0.0001
**SII baseline**									
< 730	209	153	18.7 (14.7–22.8)	1.00		99	43.6 (35.3–52.1)	1.00	
≥ 730	126	107	6.3 (5.5–8.9)	1.84 (1.43–2.36)	< 0.0001	94	13.5 (9.8–18.5)	2.36 (1.78–3.14)	< 0.0001

Abbreviations: CI, Confidence Interval; ECOG, Eastern Cooperative Oncology Group; HR, Hazard Ratio; IMDC, International Metastatic Renal Cell Carcinoma Database Consortium; MSKCC, Memorial Sloan Kettering Cancer Center; n., number; OS, Overall Survival; PFS, Progression Free Survival; pts, patients; PS, Performance Status, SII, Systemic Immune Inflammation Index.

### Changes in SII at week 6 and clinical outcome

**Figure 2:**
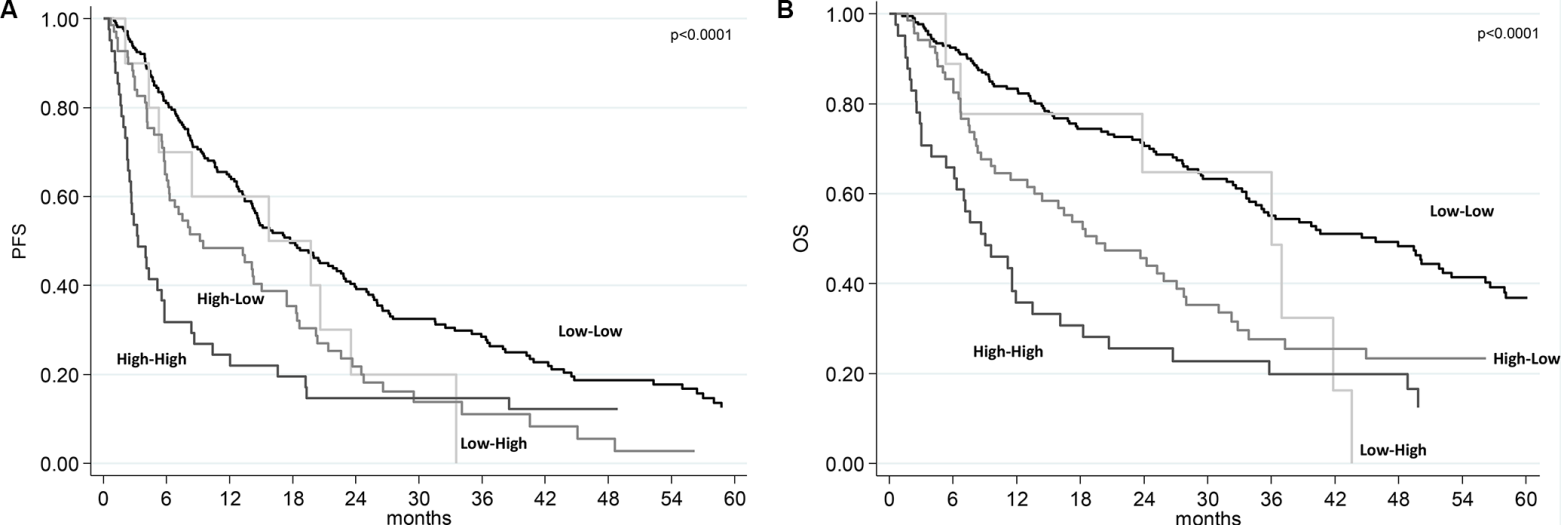
Progression-free survival (PFS) and overall survival (OS) according to SII risk group (**A**) Kaplan-Meier plots illustrating PFS according SII risk group. (**B**) Kaplan-Meier plots illustrating OS according to SII risk group.

We divided the two baseline SII groups (SII < 730 or ≥ 730) on the basis of the week 6 SII (< 730 or ≥ 730), obtaining 4 subgroups: 1) low-low (baseline SII < 730 and week 6 SII < 730); low-high (baseline SII < 730 and week 6 SII ≥ 730); high–low (baseline SII ≥ 730 and week 6 SII < 730); and high–high (baseline SII ≥ 730 and week 6 SII ≥ 730). Patients with high baseline SII that remained ≥ 730 at week 6 (high-high SII group) had a poor prognosis with a median PFS of 4 months (95% CI 2.6–5.8) and a median OS of 9.4 months (95% CI 6.1–13.5). Patients with high-low SII group had a median PFS of 9.2 mo (95% CI 6.2–10.6) and a median OS of 18.2 months (95% CI 13.1–27.1). The low-high SII group was under-represented with only 12 patients (3.6%). A better median PFS and OS were registered in patients with low SII at baseline that remained low at week 6 (18.7 and 49.4 months, respectively) (Table 3). Figure 2 shows the PFS and OS according to these four groups.

**Table 3:** Change in systemic immune-inflammation index and clinical outcome

			Progression-free survival			Overall survival		
Baseline SII	6-week SII	n. pts*	Median PFS (95% CI)	HR (95% CI)	*p*	Median OS (95% CI)	HR (95% CI)	*p*
**Low**	**Low**	197	18.7 (14.6–22.9)	1.00		49.4 (35.3–56.6)	1.00	
**Low**	**High**	12	19.7 (4.3–25.2)	1.44 (0.76–2.74)	0.271	36.0 (5.3–41.8)	1.93 (0.93–4.00)	0.075
**High**	**Low**	80	9.2 (6.2–10.6)	1.72 (1.29–2.31)	0.0004	18.2 (13.1–27.1)	2.12 (1.52–2.95)	< 0.0001
**High**	**High**	44	4.0 (2.6–5.8)	2.12 (1.48–3.04)	< 0.0001	9.4 (6.1–13.5)	3.17 (2.14–4.69)	< 0.0001

Abbreviations: CI, confidence interval; HR, hazard ratio; n., number; PFS, progression-free survival; pts, patients; OS, overall survival; SII, systemic immune-inflammation index.

* Two patients were excluded of the analysis since they had a progression or death within the 6-week (landmark analysis).

## DISCUSSION

The link between inflammation and cancer has been widely investigated in the last decades. Immune cells play a predominant role in the inflammatory process leading to the production of cytokines and chemokines that promote tumor growth, angiogenesis and metastasis [7]. Consequently, the complex balance between inflammatory cells and substances produced by inflammation may influence the type of cells detectable in the peripheral circulation. Platelets can induce circulating tumor cell epithelial-mesenchymal transition and promote extravasation of tumor cells [15, 16]. Neutrophils can promote adhesion and tumoral seeding through the secretion of circulating growth factors [17, 18]. Lymphocytes display a significant antitumoral role by inducing cytotoxic cell death and inhibiting tumor cell proliferation and migration, instituting the host's immune response to cancer [19–20]. Therefore, inflammation deeply influences cancer microenvironment that supports cancer progression.

Many studies evaluated the role of prognostic scores based on peripheral inflammation cells in several tumors, in particular in RCC. Neutrophil-lymphocyte ratio is probably the most tested prognostic index and was associated with prognosis in several tumors such as breast, lung, pancreatic, colorectal, gastric, urothelial and also kidney cancers [13, 21–22]. Lymphopenia in preoperative blood count was also associated with poor prognosis in patients with RCC [23], and in elderly patients with RCC treated with sunitinib [24]. Platelets were also associated with prognosis in RCC [25, 26]. SII combines these three parameters and has been already significantly associated with prognosis in hepatocellular carcinoma and in colorectal cancer [14, 27]. Because SII evaluates parameters also included in the IMDC model such as neutrophil and platelets counts, we investigated the characteristics and the prognosis of 335 RCC patients evaluating for the first time the prognostic power of SII in this patient population. Our study presents some limitations relating to the retrospective nature of theanalysis, and the sample size, however for the first time we demonstrated that SII at baseline and its changes at week 6 are independent predictive and prognostic factors for these RCC patients underwent first-line sunitinib (Table 3, Figure 2). Interestingly, the improvement of SII value at week 6 (from ≥ 730 to < 730) was associated with a better prognosis (Table 3), as a possible effect of sunitinib in the counts of peripheral blood cells secondary to a reduction of inflammation processes. The low-high SII group was under-represented with only 12 patients (3.6%), so no firm conclusion can be drawn on these patients. In other series, which analyzed changes on neutrophil to lymphocyte ratio the percentage with low-high group was between 0.8 to 4.8% [8, 21], so the conversion from good to bad group of systemic inflammatory markers after one cycle is confirmed to be uncommon. In clinical practice, a baseline SII value ≥ 730 decreasing at week 6 (after the first cycle of sunitinib) to value < 730, may encourage the physician to continue the treatment. In addition, we also saw a correlation between baseline and 6-week SII with clinical response/progression. Therefore, the integrated use of SII and imaging tools might lead to a significant improvement in therapeutic monitoring of patients with RCC, even if prospective study are needed to investigate this hypothesis.

The use of validated prognostic indices is essential in clinical practice to better make correct decisions on the use of high-cost drugs and to potentially reduce the impact of toxicities especially in more frail patients. In advanced RCC, the IMCD model is currently accepted as the reference in prognostic stratification and replaced in clinical practice the MSKCC criteria. In this paper we want to purpose SII as a new tool to define outcome stratification in renal cancer patients. SII changes could be able to predict response to treatment and clinical outcome of these patients, giving a potential simple tool to monitor the effect of treatment on the clinical outcome of these patients. In addition, this inflammatory index is of special interest in RCC, which is an immune-responsive disease, and new immune-oncologic agents, like checkpoint inhibitors are in active development as agents for the treatment of systemic disease [28].

In conclusion, to our knowledge, this is the first study to demonstrate that SII and its changes during treatment with sunitinib, could represent an independent prognostic factor for patients with advanced RCC undergoing first-line treatment with sunitinib. Validation in a larger prospective data set is warranted.

## MATERIALS AND METHODS

### Study patients

We retrospectively evaluated 335 patients with advanced renal cell carcinoma treated with sunitinib as first-line therapy between January 2006 and December 2014 in our seven Institutions. The SII is a new index based on platelets, neutrophils and lymphocytes counts and was defined as follows: SII = P × N/L. The X-tile 3.6.1 software (Yale University, New Haven, CT) was used for bioinformatic analysis of the baseline data to determine the cutoff value of SII.

The accuracy of all of the clinical, pathologic, and radiologic data obtained from the institutional databases was validated for each patient by an independent observer using the medical records. Data were collected into electronic data files by the local physicians and checked at the central data management. Patients with history of other treatments before sunitinib were not considered for the analysis. Sunitinib was administered according to clinical practice at the initial dose of 50 mg day with the standard schedule 4 weeks on, 2 weeks off. Dose adjustments were adopted as needed case by case according to toxicities or other relevant medical conditions. Patients were treated until disease progression or unacceptable toxicity occurred. Toxicities were evaluated and registered according to the National Cancer Institute Common Toxicity Criteria version 3.0 (NCI-CTC v.3.0). The response to treatment was assessed according to the Response Evaluation Criteria in Solid Tumors (RECIST) criteria on the basis of the validated reports obtained from the medical records. According to the clinical practice, patients were evaluated at each cycle for possible toxicities with clinical visit and full blood examinations, including a complete blood count, whereas a computed tomographic scan was done at baseline and repeated every 3 months during treatment with sunitinib. This study was carried out in accordance with the approval of the ethical committees.

### Statistical analysis

Data were summarized by frequency for categorical variables and by median and range for continuous variables. Association between categorical variables was assessed using the Chi-square or Fisher's exact test, when appropriate. Differences were considered statistically significant when *p* < 0.05. PFS was calculated from the start of first-line treatment until disease progression or last follow-up. OS was calculated from the start of first-line treatment until death or last follow-up. The Kaplan–Meier method was used to estimate PFS and OS. The logrank test and Cox proportional hazard regression were used to test for differences between groups. After univariate analysis, a multivariate analysis was carried out by Cox regression model. Estimated hazard ratios (HR), their 95% confidence intervals (95% CI), and *p* values were calculated from the Cox proportional hazard regression models.

The impact of change on survival outcomes was evaluated by the landmark analysis at 6-weeks. For this analysis, patients with early disease progression/death or patients lost to follow-up before the landmark time were excluded. All statistical analyses were carried out with SAS statistical software, version 9.4 (SAS Institute, Cary, NC).
